# Tumor Necrosis Factor (TNF) and Chemokines in Colitis-Associated Cancer

**DOI:** 10.3390/cancers3032811

**Published:** 2011-06-27

**Authors:** Naofumi Mukaida, So-ichiro Sasakki, Boryana K. Popivanova

**Affiliations:** 1 Division of Molecular Bioregulation, Cancer Research Institute, Kanazawa University, Kakuma-machi, Kanazawa 920-1192, Japan; E-Mail: s_sasaki@staff.kanazawa-u.ac.jp (S.S.); 2 Present Address, Division of Cellular Signaling, Institute for Advanced Medical Research, Keio University School of Medicine, 35 Shinanomachi, Shinjuku-ku, Tokyo 160-8582, Japan; E-Mail: popivanova_bk@a5.keio.jp

**Keywords:** cyclooxygenase 2, tumor-associated macrophage, neovascularization, NF-κB, hypoxia inducible factor

## Abstract

The connection between inflammation and tumorigenesis has been well established, based on a great deal of supporting evidence obtained from epidemiological, pharmacological, and genetic studies. One representative example is inflammatory bowel disease, because it is an important risk factor for the development of colon cancer. Moreover, intratumoral infiltration of inflammatory cells suggests the involvement of inflammatory responses also in other forms of sporadic as well as heritable colon cancer. Inflammatory responses and tumorigenesis activate similar sets of transcription factors such as NF-κB, Stat3, and hypoxia inducible factor and eventually enhances the expression of inflammatory cytokines including tumor necrosis factor (TNF) and chemokines. The expression of TNF and chemokines is aberrantly expressed in a mouse model of colitis-associated carcinogenesis as well as in inflammatory bowel disease and colon cancer in humans. Here, after summarizing the presumed actions of TNF and chemokines in tumor biology, we will discuss the potential roles of TNF and chemokines in chronic inflammation-associated colon cancer in mice.

## 1. Introduction

**Figure 1.**
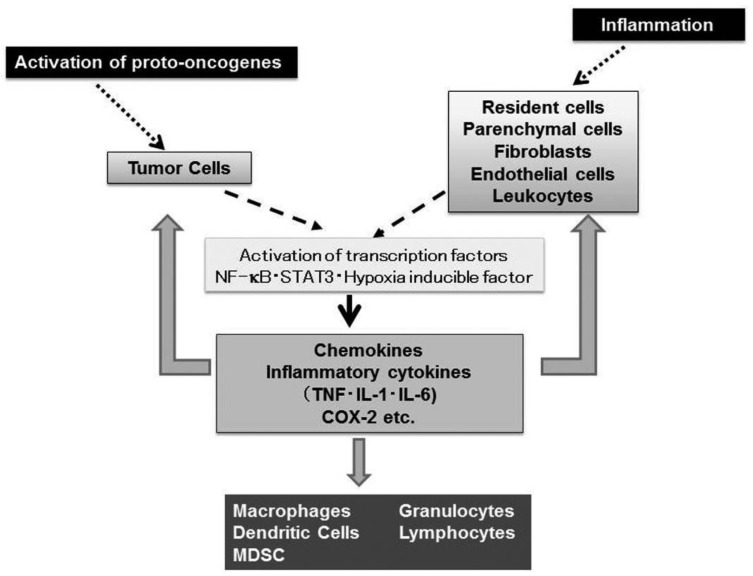
Pathways shared by tumorigenesis and inflammation

Nearly 150 years ago, Rudolf Virchow, the father of modern pathology, proposed a connection between inflammation and cancer, based on his observation that leukocytes are abundantly present in tumor tissues [1]. This assumption is supported by accumulating evidence to indicate that tumorigenesis and inflammatory responses activate similar sets of transcription factors such as NF-κB, Stat3, and hypoxia inducible factor (HIF) and eventually enhance the expression of inflammatory cytokines and chemokines [2] (Figure 1).

Virchow's view is further strengthened by histological observations that leukocytes are localized in both the tumor-supporting stroma and the tumor areas, and account for up to 50% of the tumor mass, the most predominant subset being macrophages [3]. Tumor-associated macrophages (TAM) derive mostly from circulating monocytes which are attracted into tumor sites, by locally produced chemotactic factors, such as CCL2, CCL5, CCL7, CCL8, CXCL12, vascular endothelial growth factor (VEGF), and macrophage colony stimulating factor (M-CSF) [3]. They produce various growth factors such as VEGF, transforming growth factor (TGF)-β, and fibroblast growth factor (FGF) and promote tumor development and progression [3].

Accumulating evidence indicates that a wide variety of cytokines and chemokines are produced by tumor cells as well as TAM. The production of cytokines and chemokines is induced in tumor tissues by hypoxic conditions, which tumor tissues frequently suffer from [4]. Among cytokines, tumor necrosis factor (TNF) is a major mediator of inflammation with multiple effects on various types of cells such as endothelial cells, fibroblasts, and inflammatory cells [5]. TNF can induce tissue remodeling and stromal development, which are required for tumor growth and spread, and its expression is enhanced in various types of cancers including ovarian, breast, prostate, and bladder, and colorectal cancers [5]. Likewise, a variety of chemokines have been detected in neoplastic tissues as products of either tumor cells and/or tumor stromal elements [6]. Chemokines regulate the directional movement of non-leukocytic cells as well as leukocytes [6]. Moreover, chemokines can directly affect cancer cell survival, tumor metastasis and tumor neovascularization [6,7].

Here, we will summarize the actions of TNF and chemokines in tumor biology and discuss their potential contribution to colitis-associated carcinogenesis.

## 2. TNF in Tumor Biology (Figure 2)

In response to a wide variety of activating stimuli, numerous cells synthesize the precursor form of TNF and display it as a 26-kDa type II transmembrane precursor on the plasma membrane, with the *N*-terminus in the cytoplasm and the *C*-terminus exposed to the extracellular space [8]. The TNF precursor is proteolytically cleaved between alanine (−1) and valine (+1), mainly by the action of the TNF converting enzyme (TACE), a matrix metalloproteinase (MMP)-like enzyme [8]. The resultant proteolysis leads to the formation of biologically active 17-kDa mature TNF that forms a trimer in solution. TNF exerts its pleiotropic activities after binding two related but distinct receptors, TNF receptor (TNF-R)p55/TNFR1 and TNF-Rp75/TNFR2 [9]. Both are type I transmembrane glycoproteins and exist as a trimer, but TNFR1 has a much wider distribution than TNFR2 [8,9]. Their extracellular domains consist of multiple cysteine-rich repeats and show sequence similarity with each other.

TNFR1 activation leads to recruitment of intracellular adaptor proteins that activate multiple signal transduction pathways including FAS-associated signals *via* death domain (FADD)/caspase 8/caspase 3, mitogen activated kinase (MAPK), Jun kinase (JNK)/activation protein (AP)-1, and NF-κB pathways [10]. TNFR2 singnaling leads to the activation of MAPK, JNK/AP-1, and NF-κB but not FADD/caspase 8/ caspase 3 [10]. The activation of MAPK, JNK/AP-1, and NF-κB eventually induce the expression of various molecules including interleukin (IL)-1, IL-6, chemokines, adhesion molecules, cyclooxygenase (COX)-2, and MMP (Figure 1). The activation of FADD/caspase 8/caspase 3 pathway can induce apoptosis. However, apoptosis is a late response to TNF and TNF-mediated NF-κB activation can counteract apoptosis by inducing negative regulators of apoptosis such as BCL-2 and superoxide dismutase [11].

TNF was initially identified as a factor responsible for hemorrhagic necrosis in tumor tissues in mouse [12]. TNF can induce the cell death of various tumor cells only in the presence of RNA synthesis inhibitors or protein synthesis inhibitors [12]. Subsequent characterization of TNF revealed it to be identical to an independently identified cytokine, cachectin, which can cause cachexia [13].

TNF can inhibit the function of αvβ3 integrin, an adhesion molecule expressed on tumor endothelial cells and sever the interaction between endothelial cells and the surrounding extracellular matrix. The loss of this support leads to the apoptosis of endothelial cells and hemorrhagic necrosis of tumor tissues [14]. Thus, systemic administration of a high dose of TNF induces hemorrhagic necrosis of syngeneic and xenografted tumors in mice [15]. Subsequent phase I and phase II clinical trials, however, demonstrated that systemic administration of TNF was associated with severe toxicity including cytokine storm but caused little or no tumor necrosis [16,17]. This led to the proposal that the local administration of TNF would be more successful than systemic treatment.

**Figure 2.**
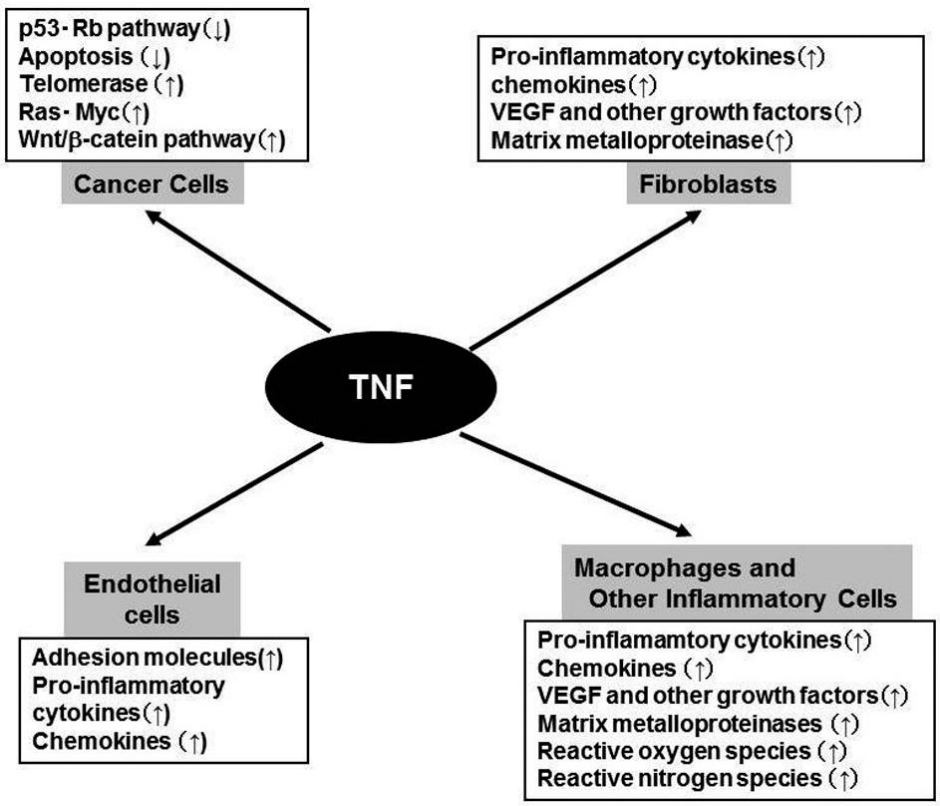
Biological activities of TNF in tumor biology.

Constitutive TNF-α expression is detected in the tumor microenvironment of many cancers, raising the possibility that it might actually be enhancing cancer growth [5,18]. Moreover, plasma TNF levels are increased in some cancer patients, especially those with a poor prognosis [5,18]. Furthermore, it became evident that TNF can induce angiogenesis [19], an indispensable step for tumor growth and metastasis, by inducing the expression of various molecules involved in angiogenesis, including MMP, COX-2, IL-1, IL-6, stromal cell-derived factor (SDF-1/CXCL12), monocyte chemoattractant protein-1 (MCP-1/CCL2), and VEGF [20](Figure 2). This cytokine network can further induce the accumulation of TAM, which are a rich source of various growth factors, particularly VEGF [21]. TNF can cause the differentiation of myeloid progenitor cells into endothelial cells in the tumor microenvironment [22]. Collectively, these observations indicate the crucial contribution of intratumoral TNF-α to tumor neovascularization.

In addition to its effects on leukocyte infiltrate and endothelial cells, TNF can directly contribute to oncogene activation and DNA damage (Figure 2). Immortalized mouse 3T3 cells can form tumors in mice, after the cells are treated with TNF for a long time [23]. TNF can induce the development of squamous cell type-like tumors from normal human epidermal cells, by activating the JNK pathway and oncogenic Ras [24]. Moreover, TNF exposure can augment the expression of spermine oxidase (SMO/PAOh1), an enzyme which oxidizes spermine into spermidine, 3-aminopropanal, and H_2_O_2_. TNF enhances the production of reactive oxygen species (ROS), with a concomitant increment in the production of 8-oxo-deoxyguanosine, a marker for oxidative DNA damage, in human lung bronchial epithelial cells [25]. Furthermore, TNF can induce the DNA and RNA editing enzyme, activation-induced cytidine deaminase (AID), in biliary cancer cells and aberrant expression of AID results in the generation of somatic mutations in tumor-related genes, including p53, c-myc, and the promoter region of the INK4A/p16 sequences [26]. Finally, TNF can induce the translocation to nucleus of the human telomerase catalytic subunit bound to NF-κB p65, thereby promoting elongation of telomere sequences, an essential step for immortalization of cells [27].

Probably due to these pro-tumorigenic activities, TNF-deficient or TNF receptor-deficient mice are resistant to carcinogenic stimuli. 7,12-Dimethylbenz[a] anthracene (DMBA) and 12-O-tetradecanoylphorbol-13-acetate (TPA) are widely used as an initiator and a promoter of skin carcinogenesis, respectively. In skin carcinogenesis induced by the combined treatment with DMBA and TPA, TNF is extensively induced in the epidermis. DMBA treatment induces c-Ha-ras mutations with similar frequencies in both wild-type (WT) and TNF-deficient mice [28]. TNF-deficient mice exhibit similar rates of malignant progression but develop fewer numbers of tumors, compared with WT mice [28]. Moreover, TNFR1-deficient and to a lesser degree, TNFR2-deficient mice develop fewer numbers of skin tumors than WT mice, when they are treated sequentially with DMBA and TPA [29]. These observations would indicate that the TNF-TNFR1 or the TNF-TNFR2 interactions are important to the early stages of tumor promotion in skin carcinogenesis.

Pre-treatment of tumor cells or mice with TNF increases the metastatic activity of transplantable tumor cells [30]. This may arise from the capacity of TNF to stabilize the protein Snail, the transcription factor that represses E-cadherin expression, the step which is required for epithelial-mesenchymal transition crucially involved in metastasis [31]. We also observed that intrasplenic injection of mouse colon cancer cells caused massive liver metastasis, depending on the presence of TNFR1. We further demonstrated that endogenously produced TNF enhanced the expression of an adhesion molecule, vascular cell adhesion molecule (VCAM)-1, on sinusoidal endothelial cells and that the enhanced VCAM-1 expression could facilitate extravasation of cancer cells and subsequent formation of metastasis foci in a TNFR1-dependent manner [32]. Collectively, TNF-α can contribute to the carcinoma development and the metastasis process.

## 3. Chemokines in Tumor Biology (Figure 3)

Chemokines are a superfamily of heparin-binding proteins with cysteine residues at their well-conserved positions [7]. Chemokines are classified into four subfamilies (designated CC, CXC, CX3C, and C) based on the location of conserved cysteine residues near their amino-terminus and most of them belong to CC or CXC subfamilies [33]. The first two cysteine residues are adjacent in the CC subfamily, while the first two cysteine residues are separated by one non-conserved amino acid in the CXC subfamily. The CXC chemokines are further grouped on the basis of the presence or the absence of another three amino acid sequence, glutamic acid-leucine-arginine (the “ELR” motif), immediately in front of the CXC sequence [7].

Evidence is accumulating to indicate that activation of several oncogenes can enhance the expression of chemokines and their receptors (Figure 1). One prominent example is that components of Ras-Raf signaling pathway induce the production of CXCL8 by activating NF-κB [34]. Constitutive activation of NF-κB induces the expression of chemokines including CXCL1, CXCL8, and CCL2 in various types of tumors [2,7]. Moreover, the tyrosine kinase, RET (Rearranged during transfection protooncogene), a prototypic transforming oncogene in human papillary carcinoma of thyroid, provokes the expression of CCL2, CCL20, angiogenic CXC chemokines, and CXC12 and its receptor, CXCR4 [35]. The CXCR4 gene can further be transactivated by the activation of HIF-1α, arising from either loss of von-Hippel-Lindau tumor suppressor (VHL) or due to the hypoxic conditions observed frequently in tumor tissues [36].

Chemokines have historically been associated to leukocyte recruitment in various conditions including tumor tissues [6]. Major attractants of monocytic cells are the CC chemokines and in a variety of cancers, CCL2 and CCL5 levels are highly correlated with high numbers of intra-tumor myeloid cells, particularly TAM [37]. TAM exhibit several characteristics of M2-polarized macrophages, consisting of an IL-12^low^IL-10^high^ phenotype, high production of prostaglandins, transforming growth factor (TGF)-β, and indoleamine dioxigenase (IDO) [38]. Thus, TAM may promote tumor progression by suppressing adaptive anti-tumor immunity. Indeed, high TAM numbers are correlated with poor patient prognosis in most human tumors, while decreased numbers of TAM is associated with decreased rates of tumor growth and fewer metastasis in mouse experimental tumor system [39]. Intratumoral chemokines can attract regulatory T cells (Treg), thereby further suppressing anti-tumor immunity [40].

Anti-tumor responses are attributable to several types of cells infiltrating into tumor site, such as tumor infiltrating lymphocytes (TIL) and dendritic cells [41]. An abundance of TIL, is a strong prognostic factor in various human cancers, especially in colorectal cancer [42]. TILs express a chemokine receptor, CXCR3 and the corresponding ligands, CXCL9 and CXCL10, can elicit anti-tumor responses which correlate with increased infiltration of CD4- and CD8-positive lymphocytes [43]. Another chemokine, CXCL16, can also recruit TIL and tumors expressing high levels of CXCL16 have slower progression with infiltration of CD4- and CD8-positive lymphocytes [44]. The pressure of apoptotic cells increases the numbers of dendritic cells migrating to the draining lymph nodes and eventually generates a specific cytotoxic T lymphocyte population by utilizing the CCL3-CCR5/CCR1 axis [45].

Neovascularization is crucial for tumor growth, progression, and metastasis [46]. Chemokines have critical roles in neovascularization in many physiological and pathological conditions, particularly tumorigenesis [7]. The ELR-positive CXC chemokines, CXCL1, CXCL2, CXCL3, CXCL5, CXCL6, CXCL7, and CXCL8 promote the migration and proliferation of endothelial cells and eventually neovascularization, mainly interacting with CXCR2, but not CXCR1 [47]. Although CXCL12 is not an ELR-positive CXC chemokine, it also has potent angiogenic effects [48]. In addition, three CC chemokines, CCL2, CCL11, and CCL16 have also been implicated in tumor neovascularization [49-51]. Indeed, CCR2, a specific receptor for CCL2, is expressed by endothelial cells and CCL2 exerts its angiogenic activity in a membrane type 1 (MT1)-MMP-dependent manner [49]. In contrast, CXCL4 and interferon-inducible ELR-negative CXC chemokines such as CXCL9, CXL10, and CXCL11 inhibit the angiogenesis induced by ELR-positive CXC chemokines, VEGF, and basic fibroblast growth factor (bFGF) [52,53]. The anti-angiogenic effects of these chemokines are mediated by a common receptor, CXCR3. In addition, Duffy antigen, which can sequester the ELR motif-positive CXC chemokines without eliciting any intracellular signals, has been shown to modulate the angiogenic effects of the ELR-motif positive CXC chemokines [54]. These observations suggest that the balance between pro-angiogenic and anti-angiogenic chemokines may determine the degree of tumor neovascularization. In addition to endothelial cells, chemokines can activate non-leukocytic cells including fibroblasts and hepatic stellate cells, which are a source of growth factors [6].

Chemokines can serve as cues for the secondary localization of tumor cells. CXCR4 is the most frequently over-expressed chemokine receptor on tumor cells, especially on cancer stem cells, and is associated with metastasis to distant organs and/or aggressive disease in breast cancer, glioblastoma, and pancreatic cancer [55-57]. In addition, many additional chemokine receptors are implicated in the malignant local invasion and/or the malignant dissemination to distant organs and lymph nodes in various types of tumors [55]. Given an inverse chemokine gradient in tumor tissues (higher levels of chemokines in tumor tissues compared to surrounding tissues), it is likely that chemokines expressed locally in tumor tissues are more important to mobilize tumor cells rather than guiding them at distant site.

## 4. TNF and Chemokines in Inflammation-associated Colon Carcinogenesis

In ulcerative colitis (UC) patients, dysplasia with nuclear atypia and loss of differentiation can be present in multiple sites of colonic mucosa [58]. The lesion is characterized by DNA damage with microsatellite instability and can progress to carcinomas [58]. Historically, the risk of cancer is 20- to 30-fold higher in patients with pancolitis of 10 or more years' duration than in a control population. However, recent progress in the treatment of UC can effectively control the disease activities of UC and eventually has reduced the frequency of colon cancer complication in UC. The trends in cancer incidence in UC patients would indicate that colon carcinoma can arise in humans at sites of chronic inflammation.

Ingestion of dextran sulfate sodium (DSS) solution causes ulceration extending continually from the rectum to distal colon in mouse and rat [59], the pathological changes mimicking those in the acute phase of UC patients. Of interest is that repeated DSS ingestion alone can induce colon carcinoma in a small proportion of mice, when the ingestion is of seven days' duration and repeated nine times [61], indicating that the inflammatory response alone can cause colon carcinoma. Azoxymethane (AOM) can cause abnormal crypt formation by inducing the generation of O^6^-methyl guanine in chromosomal DNA, when it is injected into rodents systemically. A prior administration of AOM can accelerate and increase the incidence of DSS-induced colon carcinogenesis as evidenced by the remarkably high incidence of colon cancer after only three subsequent rounds of DSS ingestion [61]. Hence, this model is frequently used as a model of chronic inflammation-associated carcinogenesis.

In this carcinogenesis model, WT mice exhibit bloody diarrhea and severe body weight loss, every time when they receive DSS [62,63]. Moreover, edema and hyperemia become evident in the middle to distal colon even after the first round of DSS intake and multiple tumors ensue in the same region after the second round of DSS intake. Histological analysis consistently demonstrates massive infiltration of leukocytes into the mucosa and edema of the submucosa, with loss of entire crypts and surface epithelium after the first round of DSS intake, particularly in the middle to distal colon. Mucosal inflammatory cell infiltration persists and is accompanied by dysplastic glands with hyperchromatic nuclei, dystrophic goblet cell appearance, and decreased mucin production. By the end of the second round of DSS intake, macroscopically visible adenocarcinomatous lesions develop, and their sizes and numbers increase progressively, thereafter. Tumor cells in adenocarcinomatous lesion are positive for cytokeratin-20, a marker of adenocarcinoma cells, with nuclear β-catenin accumulation [62].

Greten and colleagues demonstrated that in this caricinogenesis model, enterocyte-specific gene deletion of *IKKβ*, a serine/threonine kinase crucially involved in canonical NF-κB pathway activation, increased epithelial cell apoptosis and reciprocally reduced tumor incidence without few effects on inflammatory responses [63]. In contrast, deletion of *IKKβ* gene in myeloid cells attenuated inflammatory responses and diminished expression of proinflammatory cytokines including TNF, IL-1, IL-6, and CXC chemokines, without affecting apoptosis while it resulted in a significant decrease in tumor size [63]. Thus, NF-κB activation can link inflammation to cancer, by regulating the functions of inflammatory cells. However, Greten and colleagues did not identify the molecule(s), which is regulated by NF-κB and has an indispensable role in this colon carcinogenesis.

NF-κB activation can induce TNF expression and TNF is a potent activator of NF-κB [8]. These observations prompted us to investigate TNF expression in AOM+DSS-induced colon carcinogenesis. TNF mRNA was faintly expressed in untreated mice, and AOM treatment alone did not enhance TNF mRNA expression, but subsequent DSS intake augmented TNF mRNA expression [62]. Moreover, TNF protein was detected mainly in mononuclear cells present in the lamina propria and submucosal region. TNF was similarly observed in the colons of patients with active ulcerative colitis and advanced colorectal cancer, but not in normal mucosa. Furthermore, TNFR1, the major receptor for TNF, was predominantly expressed by leukocytes infiltrating the lamina propria and submucosal regions of the colon during the course of this colon carcinogenesis model [62]. Thus, the TNF-TNFR1 axis might be involved in this colon carcinogenesis, at least partly in an autocrine manner.

Indeed, TNFR1-deficient mice had less body weight loss compared with WT mice, without exhibiting bloody diarrhea even after DSS intake. Moreover, mucosal inflammatory cell infiltration and dysplastic changes of glands, but not apoptotic processes were attenuated in TNFR1-deficient mice, during the entire course of this colon carcinogenesis, compared with WT mice. Even after three rounds of DSS intake, TNFR1-deficient mice developed remarkably fewer numbers of adenocarcinomatous lesions in the colon [62]. These observations suggest a crucial role of TNFR1-mediated signals in the development of chronic inflammation-associated colon carcinogenesis process. The analysis using bone marrow chimeric mice revealed that TNFR1-mediated signals acted mainly on bone marrow-derived cells, but not non-bone marrow-derived cells, in this carcinogenesis model.

DSS ingestion induced the expression of COX-2, an enzyme that can generate prostaglandin E_2_. Prostaglandin E_2_ can induce neovascularization and activation of the Wnt/β-catenin pathway [64,65], the steps crucially involved in colon carcinogenesis. COX-2 protein was mainly detected in F4/80-positive macrophages and to a lesser extent in Ly-6G-positive neutrophils [62] and intracolonic COX-2-positive cell numbers were increased progressively in WT, but not TNFR1-deficient mice. Thus, the absence of TNFR1 reduced the infiltration of macrophages and neutrophils, a major source of COX-2 and eventually depressed colon carcinogenesis.

**Figure 4.**
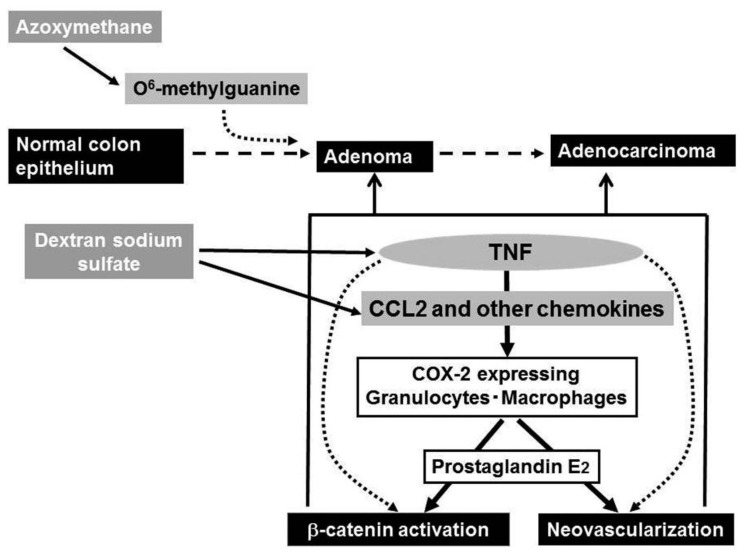
Presumed roles of TNF-α and CCL2 in AOM/DSS-induced colon carcinogenesis.

Even after the mice developed multiple colon tumors arising from the combined treatment with AOM and DSS, the treatment with a TNF antagonist, Etanercept, remarkably reduced the numbers and sizes of tumors and attenuated intracolonic infiltration by inflammatory cells, particularly neutrophils and macrophages together with a reduction in COX-2 mRNA expression and COX-2 expressing cell numbers [62] (Figure 4). Etanercept decreased the intratumoral vascular density and the nuclear accumulation of β-catenin and its mutation frequency at the tumor sites [62]. Oguma and colleagues demonstrated that infiltrating macrophage-derived TNF can directly activate Wnt/β-catenin signaling pathway in murine gastric carcinogenesis models and human gastric cancer cell lines [66]. Thus, TNF can activate the Wnt/β-catenin pathway directly and/or indirectly by enhancing COX-2 expression.

TNF has no direct chemotactic effect on macrophages, but is a potent inducer of chemokines by various types of cells [8]. Indeed, DSS treatment augments the expression of macrophage-tropic chemokines, CCL2, CCL3, and CX3CL1 [67]. In the course of this carcinogenesis process, CCL2 was detected mainly in mononuclear cells, especially macrophages infiltrating the lamina propria and submucosal regions, while it was detected also in endothelial cells at the early stages and in colon cancer cells at the later phase [68]. This may mirror the observations that CCL2 is expressed in human colorectal cancer tissues and that its expression levels correlate well with TAM numbers [37].

Intracolonic macrophages expressed CCR2 and COX-2. Moreover, mice deficient in the *CCR2* gene exhibited less macrophage infiltration and attenuated COX-2 expression when treated with the combination of AOM and DSS. Eventually, CCR2-deficient mice developed fewer colonic tumors, compared to WT mice. Moreover, CCL2 antagonists decreased intracolonic macrophage infiltration and COX-2 expression together with attenuated neovascularization, even when given after multiple colon tumors have developed [68]. Similar to the effects of a TNF antagonist, CCL2 antagonist treatment decreased the sizes and the numbers of tumors. Thus, downstream of TNF, CCL2 probably contributes to the initiation and progression of chronic colitis-associated colon carcinogenesis [67] (Figure 4).

When macrophages are exposed to hypoxia, both HIF-1α and HIF-2α accumulate in macrophages. Overexpression of HIF-2α in TAM correlates well with high-grade human tumors and poor prognosis [69]. Moreover, mice lacking *HIF-2α* gene in myeloid cells exhibit reduced macrophage infiltration and tumor development, when they are treated with the combination of AOM and DSS [69]. Concomitantly, HIF-2α modulates macrophage migration by regulating the expression of CXCR4 and M-CSF receptor expression [68]. Thus, the contribution of HIF-2α to this inflammation-associated colon carcinogenesis may be linked to another chemokine receptor, CXCR4.

Two additional inflammation-associated colon carcinogenesis models have been described. Repeated rectal administration of oxazolone can cause a chronic inflammation and prior administration of AOM can induce colon carcinogenesis [70]. This colon carcinogenesis depends on Myd88 signaling in colonic F4/80^+^CD1 1b^high^Gr1^low^ macrophages [70]. However, it remains unknown which chemokine(s) are responsible for the migration of this macrophage subset in this carcinogenesis model. Smad3-deficient mice develop mucinous adenocarcinoma by six weeks after the infection with *Helicobacter bilis* [71]. The infection augments the intracolonic expression of IL-1β and CC chemokines such as CCL3, CCL5, and CCL8 [71]. However, the pathogenic roles of these chemokines remain an open question.

## 5. Conclusions and Perspectives

TNF expression is enhanced in various types of cancer in addition to inflammation-associated colon carcinogenesis [5,18]. Given the pro-tumorigenic activities of TNF [5], phase I and/or phase II clinical trials using TNF antagonist were instituted as the treatment for breast, ovarian, and renal cancer [72-74]. TNF antagonist treatment resulted in partial remission or stabilization in 20% of patients with advanced cancer. Similarly, the crucial role of CCL2 in the recruitment of TAMs promoted the studies using anti-CCL2 antibody against a human prostate cancer xenograft model [75]. The antibody effectively induced *in vivo* tumor regression similar as observed by us in this colitis-associated carcinoma model. Thus, the treatment targeting TNF or chemokines, especially CCL2, may be effective against various cancers as well as inflammation-associated colon carcinoma.

When a large quantity of TNF or chemokines such as CCL2 is administered locally into tumor sites, for example, by gene therapy, TNF or CCL2 can provoke anti-tumor responses [76,77]. These apparent discrepancies can be explained by the assumption that tumor tissues produce low levels of TNF or chemokines, which favor tumor progression [5,6]. In contrast, when TNF or chemokines are administered into tumor tissues at far above their endogenous levels, TNF and chemokines can induce the necrosis of endothelial cells and the trafficking of immune cells, respectively, and eventually elicit anti-tumor responses. Thus, TNF or chemokines exert conflicting actions in tumor biology, in a context-dependent manner. Thus, in order to further advance TNF or chemokine antagonist treatment as anti-cancer therapy, it may be mandatory to have a clearer understanding of the roles of malignant and stromal cell-derived TNF and chemokines in human cancers under various conditions.

## Figures and Tables

**Figure 3.**
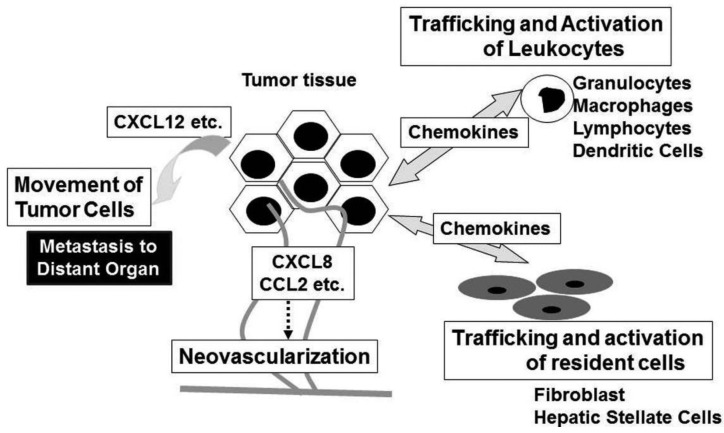
Biological activities of chemokines in tumor biology.

## References

[1] Balkwill F., Mantovani A. (2001). Inflammation and caner: back to Virchow?. Lancet.

[2] Hold G.L., El-Omar M. (2008). Genetic aspects of inflammation and cancer. Biochem. J..

[3] Sica A., Allavena P., Mantovani A. (2008). Cancer-related inflammation: The macrophage connection. Cancer Let..

[4] Allen M., Jones J.L. (2011). Jekyll and Hyde: The role of the microenvironment on the progression of cancer. J. Pathol..

[5] Balkwill F. (2009). Tumour necrosis factor and cancer. Nat. Rev. Cancer.

[6] Allavena P., Germano G., Marchesi F., Mantovani A. (2011). Chemokines in cancer related inflammation. Exp. Cell Res..

[7] Keeley E.C., Mehrad B., Strieter R.M. (2011). Chemokines as mediators of tumor angiogenesis and neovascularization. Exp. Cell Res..

[8] Wang H., Czura C.J., Tracey K.J. (2003). Tumor necrosis factor. The Cytokine Handbook.

[9] Hohmann H.P., Brockhaus M., Bauerle P.A., Remy R., Kolbeck R., van Loon A.P. (1990). Expression of types A and B tumour necrosis factor (TNF) receptors is independently regulated, and both receptors mediate activation of transcription factor NF-κB. TNF-α is not needed for induction of a biological effect *via* TNF receptors. J. Biol. Chem..

[10] Liu Z.G. (2005). Molecular mechanism of TNF signaling and beyond. Cell Res..

[11] Aggarwal B.B. (2003). Signalling pathways of the TNF superfamily: A double-edged sword. Nat. Rev. Immunol..

[12] Pennica D., Nedwin G.E., Hayflick J.S., Seeburg P.H., Derynck R., Palladino M.A., Kohr W.J., Aggarwal B.B., Goeddel D.V. (1984). Human tumour necrosis factor: precursor structure, expression and homology to lymphotoxin. Nature.

[13] Beutler B., Cerami A. (1986). Cachectin and tumour necrosis factor as two sides of the same biological coin. Nature.

[14] Rüegg C., Yilmaz A., Bieler G., Bamat J., Chaubert P., Lejeune F.J. (1998). Evidence for the involvement of endothelial cell integrin αvβ3 in the disruption of the tumor vasculature induced by TNF and IFN-γ. Nat. Med..

[15] Talmadge J.E., Phillips H., Schneider M., Rowe T., Pennington R., Bowersox O., Lenz B. (1988). Immunomodulatory properties of recombinant murine and human tumor necrosis factor. Cancer Res..

[16] Selby P., Hobbs S., Viner C., Jackson E., Jones A., Newell D., Calvert A.H., McElwain T., Fearon K., Humphreys J. (1987). Tumour necrosis factor in man: Clinical and biological observations. Br. J. Cancer.

[17] Feldman E.R., Creagan E.T., Schaid D.J., Ahmann D.L. (1992). Phase II trial of recombinant tumor necrosis factor in disseminated malignant melanoma. Am. J. Clin. Oncol..

[18] Szlosarek P., Charles K.A., Balkwill F.R. (2006). Tumour necrosis factor-α as a tumour promoter. Eur. J. Cancer.

[19] Leibovich S.J., Polverini P.J., Shepard H.M., Wiseman D.M., Shively V., Nuseir N. (1987). Macrophage-induced angiogenesis is mediated by tumour necrosis factor-α. Nature.

[20] Kulbe H., Thompson R., Wilson J.L., Robinson S., Hagemann T., Fatah R., Gould D., Ayhan A., Balkwill F. (2007). The inflammatory cytokine tumor necrosis factor-alpha generates an autocrine tumor-promoting network in epithelial ovarian cancer cells. Cancer Res..

[21] Meng Y., Beckett M.A., Liang H., Mauceri H.J., van Rooijen N., Cohen K.S., Weichselbaum R.R. (2010). Blockade of tumor necrosis factor α signaling in tumor-associated macrophages as a radiosensitizing strategy. Cancer Res..

[22] Li B., Vincent A., Cates J., Brantley-Sieders D.M., Polk D.B., Young P.P. (2009). Low levels of tumor necrosis factor α increase tumor growth by inducing an endothelial phenotype of monocytes recruited to the tumor site. Cancer Res..

[23] Komori A., Yatsunami J., Suganuma M., Okabe S., Abe S., Sakai A., Sasaki K., Fujiki H. (1993). Tumor necrosis factor acts as a tumor promoter in BALB/3T3 cell transformation. Cancer Res..

[24] Zhang J.Y., Adams A.E., Ridky T.W., Tao S., Khavari P.A. (2007). Tumor necrosis factor receptor 1/c-Jun-NH2-kinase signaling promotes human neoplasia. Cancer Res..

[25] Babbar N., Casero R.A. (2006). Tumor necrosis factor-α increases reactive oxygen species by inducing spermine oxidase in human lung epithelial cells: a potential mechanism for inflammation-induced carcinogenesis. Cancer Res..

[26] Komori J., Marusawa H., Machimoto T., Endo Y., Kinoshita K., Kou T., Haga H., Itai I., Uemoto S., Chiba T. (2008). Activation-induced cytidine deaminase links bile duct inflammation to human cholangiocarcinoma. Hepatol..

[27] Akiyama M., Hideshima T., Hayashi T., Tai Y.T., Mitsiades C.S., Mitsiades N., Chaudan D., Richardson P., Munshi N.C., Anderson K.C. (2003). Nuclear factor-κB p65 mediates tumor necrosis factor α-induced nuclear translocation of telomerase reverse transcriptase protein. Cancer Res..

[28] Moore R.J., Owens D.M., Stamp G., Arnott C., Burke F., East N., Holdsworth H., Turner L., Rollins B., Pasparakis M., Kollias G., Balkwill F. (1999). Mice deficient in tumor necrosis factor-α are resistant to skin carcinogenesis. Nat. Med..

[29] Arnott C.H., Scott K.A., Moore R.J., Robinson S.C., Thompson R.G., Balkwill F.R. (2004). Expression of both TNF-α receptor substypes is essential for optimal skin tumour development. Oncogene.

[30] Orosz P., Echtenacher B., Falk W., Rűschoff J., Weber D., Männel D.N. (1993). Enhancement of experimental metastasis by tumor necrosis factor. J. Exp. Med..

[31] Wu Y., Deng J., Rychahou P.G., Qiu S., Evers B.M., Zhou B.P. (2009). Stabilization of Snail by NF-κB is required for inflammation-induced cell migration and invasion. Cancer Cell.

[32] Kitakata H., Nemoto-Sasaki Y., Takahashi Y., Kondo T., Mai M., Mukaida N. (2002). Essential roles of tumor necrosis factor receptor p55 in liver metastasis of intrasplenic administration of colon 26 cells. Cancer Res..

[33] Nomiyama H., Osada N., Yoshie O. (2010). The evolution of mammalian chemokine genes. Cytokine Growth Fact. Rev..

[34] Sparmann A., Bar-Sagi D. (2004). Ras-induced interleukin-8 expression plays a critical role in tumor growth and angiogenesis. Cancer Cell.

[35] Borrello M.G., Alberti L., Fischer A., Degl'innocenti D., Ferrario C., Gabriboldi M., Marchesi F., Allavena P., Greco A., Collini P., Pilloti S., Cassinelli G., Bressan P., Fugazzola L., Mantovani A., Pierotti M.A. (2005). Induction of a proinflamamtory program in normal human thyrocytes by the RET/PTC1 oncogene. Proc. Natl. Acad. Sci. USA.

[36] Shioppa T., Uranchimeg B., Saccani A., Biswas S.K., Doni A., Rapisarda A., Bernasconi S., Saccani A., Nebuloni M., Vago L., Mantovani A., Melillo G., Sica A. (2003). Regulation of the chemokine receptor CXCR4 by hypoxia. J. Exp. Med..

[37] Bailey C., Negus R., Morris A., Ziprin P., Goldin R., Allavena P., Peck D., Darzi A. (2007). Chemokine expression is associated with the accumulation of tumor associated macrophages (TAMs) and progression in human colorectal cancer. Clin. Exp. Metastasis.

[38] Mantovani A., Sozzani S., Locati M., Allavena P., Sica A. (2002). Macrophage polarization: Tumor-associated macrophages as a paradigm for polarized M2 mononuclear phagocytes. Trends Immunol..

[39] Condeelis J., Pollard J.W. (2006). Macrophages: Obligate partners for tumor cell migration, invasion, and metastasis. Cell.

[40] Nihishikawa H., Sakaguchi S. (2010). Regulatory T cells in tumor immunity. Int. J. Cancer.

[41] Steer H.J., Lake R.A., Nowak A.K., Robinson B.W.S. (2010). Harnessing the immune response to treat cancer. Oncogene.

[42] Laghi L., Bianchi P., Miranda E., Balldore E., Pacetti V., Grizzi F., Allavena P., Torri V., Repici A., Santoro A., Mantovani A., Roncalli M., Malesci A. (2009). CD3^+^ cells at the invasive margin of deeply invading (pT3-T4) colorectal cancer and risk of post-surgical metastasis: A longitudinal study. Lancet Oncol..

[43] Pan J., Burdick M.D., Belperio J.A., Xue Y.Y., Gerard C., Sharma S., Dubinett S.M., Strieter R.M. (2006). CXCR3/CXCR3 ligand biological axis impairs Renca tumor growth by a mechanism of immunoangiostasis. J. Immunol..

[44] Meijer J., Ogink J., Kreike B., Nuyten D., de Visser K.E., Roos E. (2008). The chemokine receptor CXCR6 and its ligand CXCL16 are expressed in carcinomas and inhibit proliferation. Cancer Res..

[45] Iida N., Nakamoto Y., Baba T., Kakinoniki K., Li Y.-Y., Wu Y., Matsushima K., Kaneko S., Mukaida N. (2008). Tumor cell apoptosis induces tumor-specific immunity in a CC chemokine receptor 1- and 5-dependent manner. J. Leukoc. Biol..

[46] Fidler I.J., Ellis E.M. (1994). The implications of angiogenesis for the biology and therapy of cancer metastasis. Cell.

[47] Addison C.L., Daniel T.O., Burdick M.D., Liu H., Ehlert J.E., Xue Y.Y., Buechi L., Walz A., Richmond A., Strieter R.M. (2000). The CXC chemokine receptor, CXCR2, is the putative receptor for ELR^+^ CXC chemokine-induced angiogenic activity. J. Immunol..

[48] Salcedo R., Oppenheim J.J. (2003). Role of chemokines in angiogenesis: CXCL12/SDF-1 and CXCR4 interaction, a key regulator of endothelial cell response. Microcirculation.

[49] Gálvez B.G., Genis L., Matias-Román S., Oblander S.A., Tryggvason K., Apte S.S., Arroyo A.G. (2005). Membrane type 1-matrix metalloproteinase is regulated by chemokines monocyte-chemoattractant protein-1/ccl2 and interleukin-8/CXCL8 in endothelial cells during angiogenesis. J. Biol. Chem..

[50] Salcedo R., Young H.A., Ponce M.L., Ward J.M., Kleinman H.K., Murphy W.J., Oppenheim J.J. (2001). Eotaxin (CCL11) induces in vivo angiogenic responses by human CCR3+ endothelial cells. J. Immunol..

[51] Strasly M., Doronzo G., Cappello P., Valdembri D., Arese M., Mitola S., Moore P., Alessandri G., Giovarelli M., Bussolino F. (2004). CCL16 activates an angiogenic program in vascular endothelial cells. Blood.

[52] Mainoen T.E., Gray G.S., Petro J., Hunt A.J., Donner A.L., Bauer S.I., Carson H.F., Sharpe R.J. (1990). Inhibition of angiogenesis by recombinant human platelet factor-4 and related peptides. Science.

[53] Romagnani P., Annunziato F., Lasagni L., Lazzeri E., Beltrame C., Francalanci M., Uguccinioni M., Galli G., Cosmi L., Maurenzig L., Baggiolini M., Maggi E., Romagnani S., Serio M. (2001). Cell-cycle-dependent expression of CXC chemokine receptor 3 by endothelial cells mediates angiostatic activity. J. Clin. Invest..

[54] Du J., Luan J., Liu H., Daniel T.O., Peiper S., Chen T.S., Yu Y., Horton L.W., Nanney L.B., Strieter R.M., Richmond A. (2002). Potential role for Duffy antigen chemokine binding-protein in angiogenesis and maintenance of homeostasis in response to stress. J. Leukoc. Biol..

[55] Műller A., Homey B., Soto H., Ge N., Catron D., Buchanan M.E., McClanahan T., Murphy E., Yuan W., Wagner S.N., Barrera J.L., Mohar A., Zlotnik A. (2001). Involvement of chemokine receptors in breast cancer metastasis. Nature.

[56] Zhou Y., Larsen P.H., Hao C., Yong V.W. (2002). CXCR4 is a major chemokine receptor on glioma cells and mediates their survival. J. Biol. Chem..

[57] Hermann P.C., Huber S.L., Herrler T., Aicher A., Ellwart J.W., Guba M., Bruns C.J., Heeschen C. (2007). Distinct populations of cancer stem cells determine tumor growth and metastatic activity in human pancreatic cancer. Cell Stem Cell.

[58] Abraham C., Cho J.H. (2009). Inflammatory bowel disease. N. Engl. J. Med..

[59] Okayasu I., Hatakeyama S., Yamada M., Ohkusa T., Inagaki Y., Nakaya R. (1990). A novel method in the induction of reliable experimental acute and chronic ulcerative colitis in mice. Gastroenterology.

[60] Okayasu I., Yamada M., Mikami T., Yoshida T., Kanno J., Ohkusa T. (2002). Dysplasia and carcinoma development in a repeated dextran sulfate sodium-induced colitis model. J. Gastroenterol. Hepatol..

[61] Okayasu I., Ohkusa T., Kajiura K., Kanno J., Sakamoto S. (1996). Promotion of colorectal neoplasia in experimental murine ulcerative colitis. Gut.

[62] Popivanova B.K., Kitamura K., Wu Y., Kondo T., Kagaya T., Kaneko S., Oshima M., Fujii C., Mukaida N. (2008). Blocking TNF-α in mice reduces colorectal carcinogenesis associated with chronic colitis. J. Clin. Invest..

[63] Greten F.R., Eckmann L., Greten T.F., Park J.M., Li Z.W., Egan L.J., Kagnoff M.F., Karin M. (2004). IKKβ links inflammation and tumorigenesis in a mouse model of colitis-associated cancer. Cell.

[64] Seno H., Oshima M., Ishikawa T.O., Oshima H., Takaku K., Chiba T., Narumiya S., Taketo M.M. (2002). Cyclooxygenase 2- and prostaglandin E_2_ receptor EP_2_-dependent angiogenesis in Apc^Δ716^ mouse intestinal polyps. Cancer Res..

[65] Castellone M.D., Teramoto H., Williams B.O., Druey K.M., Gutkind J.S. (2005). Prostaglandin E_2_ promotes colon cancer cell growth through a Gs-axin-β-catenin signaling axis. Science.

[66] Oguma K., Oshima H., Aoki M., Uchio R., Naka K., Nakamura S., Hirao A., Saya H., Taketo M.M., Oshima M. (2008). Activated macrophages promote Wnt signalling through tumour necrosis factor-α in gastric tumour cells. EMBO J..

[67] Alex P., Zachos N.C., Nguyen T., Gonzalez L., Chen T.-E., Conklin L.S., Centola M., Li X. (2009). Distinct cytokine patterns identified from multiplex profiles of murine DSS and TNBS-induced colitis. Inflamm. Bowel Dis..

[68] Popivanova B.K., Kostadinova F.I., Furuichi K., Shamekh M.M., Kondo T., Wada T., Egashira K., Mukaida N. (2009). Blockade of a chemokine, CCL2, reduces chronic colitis-associated carcinogenesis in mice. Cancer Res..

[69] Imtiyaz H.Z., Williams E.P., Hickey M.M., Patel S.A., Durham A.C., Yuan L.-J., Hammond R., Gimotty P.A., Keith B., Simon M.C. (2010). Hypoxia-inducible factor 2α regulates macrophage function in mouse models of acute and tumor inflammation. J. Clin. Invest..

[70] Shiechl G., Bauer B., Fuss I., Lang S.A., Moser C., Ruemmele P., Rose-John S., Neurath M.F., Geissler E.K., Schlitt H.-J., Strober W., Fichtner-Feigl S. (2011). Tumor development in murine colitis depends on MyD88 signaling of colonic F4/80^+^CD11b^high^Gr1^low^ macrophages. J. Clin. Invest..

[71] Ericsson A.C., Myles M., Davis W., Ma L., Lewis M., Maggio-Price L., Franklin C. (2010). Noinvasive detection of inflammation-associated colon cancer in a mouse model. Neoplasia.

[72] Madhusudan S., Foster M., Muthuramalingam S.R., Braybrooke J.P., Wilner S., Kaur K., Han C., Hoare S., Balkwill F., Talbot D.C., Ganesan T., Harris A.L. (2004). A phase II study of etanercept (Enbrel), a tumor necrosis factor alpha inhibitor in patients with metastatic breast cancer. Clin. Cancer Res..

[73] Harrison M.L., Obermueller E., Maisey N.R., Hoare S., Edmonds K., Li N.F., Chao D., Hall K., Timotheadou E., Charles K., Ahern R., King D.M., Eisen T., Corringham R., DeWitte M., Balkwill F., Gore M. (2007). Tumor necrosis factor alpha as a new target for renal cell carcinoma: Two sequential phase II trials of infliximab at standard and high dose. J. Clin. Oncol..

[74] Brown E.R., Charles K.A., Hoare S.A., Rye R.L., Jodrell D.I., Aird R.E., Vora R., Prabhakar U., Nakada M., Corringham R.E., DeWitte M., Sturgeon C., Propper D., Balkwill F.R., Smyth J.F. (2008). A clinical study assessing the tolerability and biological effects of infliximab, a TNF-alpha inhibitor, in patients with advanced cancer. Ann. Oncol..

[75] Loberg R.D., Ying C., Craig M., Day L.L., Sargent E., Neeley C., Wonjo K., Snyder L.A., Yan L., Pienta K.J. (2007). Targeting CCL2 with systemic delivery of neutralizing antibodies induces prostate cancer tumor regression *in vivo*. Cancer Res..

[76] Hernandez J., Cooper J., Babel N., Morton C., Rosemurgy A.S. (2010). TNF-α gene delivery therapy for solid tumors. Expert Opin. Biol. Ther..

[77] Chada S., Ramesh R., Mhashilkar A.M. (2003). Cytokine- and chemokine-based gene therapy for cancer. Curr. Opin. Mol. Ther..

